# Exploring the effectiveness of the Career Guidance and Counseling Program on the perceived readiness for the job market: a lived experience among nursing students

**DOI:** 10.3389/fpubh.2024.1403730

**Published:** 2024-10-22

**Authors:** Hend Abdu Alnajjar, Ebtsam Aly Abou Hashish

**Affiliations:** ^1^College of Nursing, King Saud bin Abdul-Aziz University for Health Sciences, Jeddah, Saudi Arabia; ^2^King Abdullah International Medical Research Center, Jeddah, Saudi Arabia; ^3^Faculty of Nursing, Alexandria University, Alexandria, Egypt

**Keywords:** Career Guidance and Counseling Program, lived experience, nursing students, job market, job readiness

## Abstract

**Background:**

The current workforce demands that nursing graduates possess the necessary skills and knowledge to perform in complex clinical and professional environments. Career guidance can potentially improve students’ job readiness by increasing their confidence in career pursuits, simplifying career decisions, and helping them address decision-making challenges.

**Methods:**

This phenomenological study aimed to explore nursing students and interns’ perspectives on the effectiveness of the Career Guidance and Counseling Program (CGCP) and its impact on their readiness for the job market through their lived experiences while participating in the program. Data were collected through in-depth semi-structured interviews with a purposive sample of 28 Saudi university students and continued until data saturation was reached. Thematic analysis was used for the data analysis.

**Results:**

Seven themes emerged: personal experiences with the CGCP, the program’s importance, effectiveness, benefits, strengths, and weaknesses, and recommendations for improvement. Overall, participants expressed gratitude for the CGCP and believed that it would positively impact their future career success. They also felt that the program provided them opportunities to share ideas, information, and concerns, thereby improving their career decision-making and adaptability.

**Conclusion:**

The results show that CGCP positively affected participants’ lives by providing support, guidance, and resources for informed career decision-making, developing essential career-related skills, and navigating career transitions. These findings have practical implications for nursing colleges implementing similar programs for better integration into the curriculum and for the continuity of such vital programs to help students. Future studies should explore this topic in different colleges and specialties.

## 1 Introduction

The National Association of Colleges and Employers (NACE) identified eight fundamental competencies vital for thriving in the labor market: career development, effective communication, critical thinking, equity, leadership, professionalism, teamwork, and technological skills (1). However, studies have revealed a gap between these skills and those of college graduates, indicating a mismatch between educational outcomes and job market needs (2, 3). Career guidance services play a vital role in addressing this disparity by preparing students for career transitions and equipping them with the skills needed for success in today’s rapidly evolving work environment (4). Improving the quality and quantity of career guidance enhances students’ career readiness (5). Similarly, the United Nations Educational, Scientific and Cultural Organization (UNESCO) (6) emphasizes the role of career guidance in helping students understand their personal characteristics and make informed career choicest that align with their values, interests, and abilities (2).

In today’s academic landscape, student career guidance and counseling are indispensable components of university support services, extending beyond academic instruction to play a crucial role in shaping students’ success as professionals (7). To help students navigate the complexities of career readiness, a holistic approach that encompasses personal development and goal alignment is imperative. Career readiness involves more than simply formulating a career plan; it entails developing personal beliefs, attitudes, motivation, and the skills necessary for successful career building and ensuring they align with individuals’ aspirations and goals (7, 8). This holistic approach to career readiness underlines the importance of providing comprehensive career guidance programs to prepare students for the challenges they will face when they enter the workforce (9).

Likewise, career exploration and decision-making are fundamental to career readiness and are influenced by students’ diverse educational backgrounds, cultural experiences, and exposure to the modern world (10). Effective career guidance programs should recognize and address these contextual factors to provide tailored support to students as they navigate their career paths. As students advance through their academic journey, proactive career planning becomes crucial for professional growth. Thus, career planning and preparation are vital components in shaping students’ professional development and establishing a foundation for their future roles (11, 12). Career planning is an iterative process that involves self-assessment, goal setting, skill enhancement, and exploration of suitable career paths that benefits individuals at all stages of their nursing careers. Offering structured career guidance empowers students to make informed career decisions, thereby reducing early career challenges and mitigating burnout (11, 13). Thus, a holistic approach to career guidance involving exploration, readiness, and planning ensures that students are adequately prepared to excel in their profession. Accordingly, this study aimed to explore the effectiveness of the Career Guidance and Counseling Program (CGCP) on perceived readiness for the job market through a qualitative investigation of the lived experiences of nursing students who participated in this program.

### 1.1 Theoretical framework

**Figure 1:**
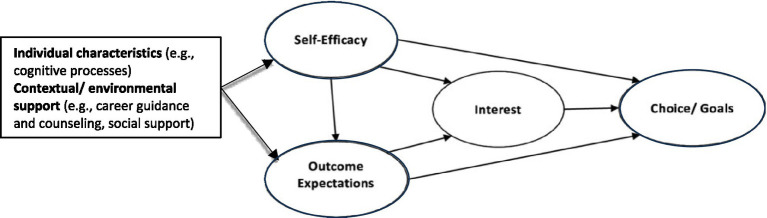
Social cognitive career theory (SCCT) framework adapted from Duong and St-Jean (17).

Social cognitive career theory (SCCT) provides a valuable framework for understanding how career guidance interventions influence students’ workforce readiness, emphasizing the interaction between individuals’ internal cognitive processes and external contextual factors (e.g., career guidance and counseling, and social support) in shaping career decisions and pathways (14) (see Figure 1). According to SCCT, feelings of competence or self-efficacy play a key role in influencing career choices and goal setting. In addition, self-efficacy beliefs and outcome expectations significantly contribute to the development of career interests (15, 16).

Empirical studies have consistently demonstrated the effectiveness of SCCT in various contexts, particularly career guidance and counseling programs. For instance, Duong and St-Jean (17) found that perceived educational support significantly influenced the development of self-efficacy and career interests among higher education students, suggesting that SCCT can effectively guide their entrepreneurial intentions. Similarly, Reed et al. (18) reported that a career development course based on cognitive information processing theory, which aligns closely with SCCT, reduces students’ negative career thoughts and improves their decision-making confidence. Wang et al. (19) investigated the impact of career and psychosocial mentoring on newcomer socialization and revealed that mentoring enhances self-efficacy, a core component of SCCT, and helps newcomers adjust more effectively to their roles. Crucially, these studies underscore the utility of SCCT in enhancing career decision-making, reducing anxiety, and fostering self-efficacy, making it an invaluable framework for career guidance programs.

Numerous contextual factors influence students’ life skills and career aspirations, including the presence of a nurturing environment, access to comprehensive guidance and counseling programs, and feelings of connection with teachers and peers (20). Positive attitudes, competencies, and the construction of self and professional identities are vital for effective career planning and overall life satisfaction (20, 21). Given this theoretical and conceptual framework, we assume that providing students with awareness and guidance through learning activities focused on career planning would enhance their career knowledge and self-readiness, ultimately preparing them to make informed career choices and to effectively transition into the job market.

### 1.2 Study aim and significance

This study aimed to explore nursing students’ perspectives on the effectiveness of the CGCP and its impact on their readiness for the job market through their lived experiences of attending such programs. To address a significant gap in understanding the specific challenges and opportunities within career education for nursing students, this study targeted limited comprehensive data regarding the perceived importance and effectiveness of current career education programs in preparing nursing students for the labor market and their professional lives.

University alumni transitioning into the workforce often face challenges that underscore the importance of career counseling services and psychological support. While theoretical knowledge and practical skills are essential in the nursing profession, transferable soft skills are equally important (7). Nursing educators, nurse managers, and alumni play vital roles in guiding students toward career opportunities and helping them make informed decisions during their education (9, 22). However, it is also crucial to identify students’ specific training needs and experiences in career guidance and counseling programs. By considering students’ values, interests, and goals, educators and mentors can help them acquire the necessary knowledge, skills, and position themselves for future career success (9).

The demanding nature of nursing highlights the importance of adequately preparing graduates for the complexities of clinical practice. However, there remains a notable lack of emphasis on structured career guidance and counseling in nursing education (23, 24). Despite efforts to foster students’ career competencies (11, 12), concerns persist regarding the preparedness of new nursing graduates in the workforce (25). Colleges can play a pivotal role in bridging this gap by offering career-oriented courses and interventions that help students map their career paths and make informed decisions (26).

Research has consistently highlighted the positive impacts of career intervention programs. Lindo et al. (23) demonstrated the progressive effects of career counseling, and Wong et al. (27) identified various forms of teacher support that are beneficial for career planning. In the nursing context, Abou Hashish and Bajbeir (9) emphasize the importance of integrating career guidance sessions into the curriculum before graduation and during internships, showing how career intervention programs help students overcome decision-making challenges and enhance career self-efficacy (28).

Our study aims to address the research gap by employing a qualitative phenomenological approach to evaluate the effectiveness of the CGCP. Specifically, this study explores how the program enhances nursing students’ educational and career aspirations, readiness for the job market, and career planning skills in preparation for the workforce. By examining students’ experiences and perspectives, this study provides valuable insights into the development of more effective career guidance interventions. Through thematic analysis, key themes that may guide future improvements to the program are uncovered and actionable feedback to university administrators is provided to enhance student outcomes.

### 1.3 Study context (background on CGCP)

Recognizing their pivotal role in preparing nursing students for the workforce, educational institutions have intensified efforts to enhance career and life-planning education (13). In response, King Saud bin Abdul Aziz University for Health Sciences (KSAU-HS) requested that each college’s alumni unit establish a CGCP program aligned with national educational and health sector goals that is tailored to its graduates’ specific needs. Consequently, under the supervision of the KSAU’s Students’ Deanship, the College of Nursing’s Alumni Unit developed a structured CGCP to guide nursing students through career self-exploration and self-management. The program’s objectives include enhancing self-understanding and goal-setting skills, cultivating career-related values, developing leadership abilities, and helping students make informed career decisions. Initially scheduled to be completed within 4 months with 10 sessions, the program was later expanded to 14 sessions based on the participants’ interests, in addition to an alumni reunion day.

The program sessions were delivered through a combination of in-person and online methods, including on-site workshops, awareness sessions, and interactive sessions via Microsoft Teams and X (Twitter). College alumni led some sessions, whereas faculty members and nurse managers led others to provide current students with opportunities to learn from their experiences. This interaction encouraged reflection on social, academic, and intellectual identities as well as preparation for future career paths. Multiple channels, including the university message center, college email distributions, personal invitations via WhatsApp groups, the alumni unit’s Twitter account, and shared program announcements, were utilized. The session duration ranged from 1 to 2 h. Attendees’ satisfaction with each session was evaluated using a simple survey on a scale of 1–5, and feedback was provided on areas for improvement. See Table 1 for the details of the program.

**Table 1:** Descriptive data of the Career Guidance and Counseling Program (CGCP).

Session title	Type of session	Presenter	Number of attendees	Average Satisfaction level/5
1. Introduction to the program (university guidance program and job market requirements, career planning and exploration process) and its content and announcement strategies	Interactive lecture and discussion	Program coordinator	55	4.6
2. After internship year: find your way and career path.	Interactive lecture and discussion	Alumni and Program coordinator	52	4.7
3. Professional development and hard-soft skills	Workshop	Program coordinator and Alumni	120	5
4. Alumni experience for preparing for Saudi nursing licensure examination (SNLE)	X: Twitter space for Interactive discussion	Alumni and Nursing intern	72	5
5. Alumni scholarship experience	Interactive lecture and discussion	Alumni and Scholarship returnee	33	4.8
6. Preparation for hiring interview and CV	Workshop	Faculty member and Alumni	50	4.8
7. Parallel thinking and six -thinking hats	Workshop	Faculty member and Alumni	70	4.6
8. Knowing your professional rights	Interactive lecture and discussion	Faculty member	33	4.7
9. Explore your personal and clinical leadership	Workshop	Faculty member	33	4.6
10. What the nurse executives expect and need from nursing graduates	Twitter space for Interactive discussion	Hospital Nurse executive and Senior nursing students	54	5
11. Socialization and networking in nursing profession	Workshop	Faculty member	34	4.5
12. How to develop and market yourself digitally	Workshop	Information technology supervisor and Program coordinator	34	5
13. Communication and patient safety	Workshop	Faculty member	35	4.5
14. Challenges and opportunities that facing nursing alumni in the workplace.	Panel discussion	Group of Alumni and Program coordinator	45	5
Alumni day celebration	Open day	Program coordinator and Alumni	250	5
Total attendees of the program sessions	805			
Average satisfaction and percentages	4.77 = 95.43%			

## 2 Research procedures/methods

### 2.1 Study design and setting

A qualitative research design utilizing a phenomenological approach as outlined by Creswell (29), was conducted at the College of Nursing, Jeddah (CONJ), KSAU-HS, in Jeddah, Saudi Arabia. Phenomenological research seeks to understand and describe the meaning of individuals’ lived experiences within a specific context (Creswell, 29). The phenomenological approach is appropriate for this study because it focuses on capturing the essence of students’ personal experiences with the CGCP to provide insights into how the program influences their preparedness for future careers. Creswell’s framework was instrumental in guiding the exploration of these lived experiences through semi-structured interviews, which allowed for a deeper understanding of the studied phenomenon.

### 2.2 Study participants and inclusion criteria

This study selected a purposive sample of senior nursing students from CONJ who participated in the CGCP during the academic year 2023–2024. Purposive sampling, a non-probability sampling technique, was employed to select participants based on specific characteristics or qualities aligned with the objectives of the study. This method is particularly useful when focusing on a subset of a population that possesses specific knowledge or experience relevant to a research topic (30). Participants had to attend at least six sessions of the program to meet the eligibility criteria; those who attended fewer than six sessions were excluded. The sampling approach was guided by Polkinghorne's (31) recommendation to interview 5–15 individuals who have experienced the phenomenon under study. Participants were selected based on their current enrollment, experience with the CGCP, and voluntary participation, thereby ensuring the relevance of the data to the study’s objectives. Participants were included until data saturation was reached, which is defined as the point at which no new information emerged during data collection (32). Data collection commenced with a pilot interview and continued until 28 interviews were conducted.

### 2.3 Data collection method

A semi-structured interview (SSI) is a qualitative data collection method that combines predetermined open-ended questions with the flexibility to explore topics that arise naturally during conversations. Unlike fully structured interviews, which strictly adhere to a set list of questions, SSIs allow the interviewer to delve deeper into issues as they emerge, making them particularly effective for understanding complex phenomena from the participants’ perspectives (33). The semi-structured interview guide consisted of two sections.

Section 1: Demographic information - this section included questions about participants’ age, academic level, the number of CGCP sessions attended, and a question for rating their perception of the program’s effectiveness, assessed on a five-point Likert scale ranging from “5 = very effective” to “1 = very ineffective.”

Section 2: Open-ended questions - this section featured broad, predetermined questions designed to elicit in-depth qualitative data. The questions were intended to allow participants to freely express their perceptions of and experiences with CGCP. The semi-structured format also enabled the interviewer to follow up with probing questions based on participants’ responses, allowing for a deeper exploration of the topics discussed. The following research questions were posed to explore nursing students’ perspectives on the effectiveness of the CGCP and its impact on their readiness for the job market. These questions were presented in a flexible order to adapt to the flow of the students’ lived experiences during the interviews. (1) What are the experiences of nursing students attending the CGCP? (2) How do students perceive the importance of CGCP before graduation? (3) How effective is the CGCP in preparing students for the job market from a student perspective? (4) What benefits do students perceive the CGCP offers in shaping their views of the job market? (5) What do nursing students perceive as the strengths and weaknesses of the CGCP? What recommendations do students have for improving the CGCP?

The interview guide was validated through a peer review to ensure its relevance and appropriateness. Additionally, two pilot interviews were conducted to refine the clarity of the questions and assess the interviewers’ proficiency in conducting the interviews. As recommended by Grove et al. (34), this preparatory process ensured that the questions were clear, and the interviewers were well prepared to engage with the participants effectively. The interviewers were knowledgeable faculty members with experience in conducting interviews and a background in qualitative research. Their expertise ensured professional conduct of the interviews, adherence to ethical standards, and a deep understanding of how to elicit meaningful responses while minimizing bias. Furthermore, they were skilled in building a rapport with the participants and maintaining confidentiality throughout the process.

### 2.4 Data collection

After obtaining approval from both the CONJ Research Committee and King Abdullah International Medical Research Center’s (KAIMRC) Institutional Review Board, the researchers conducted semi-structured interviews with the participants either face-to-face or via Microsoft Teams, based on their preferences. The researchers briefed the participants about the nature of the study, ethical considerations, expected duration, data confidentiality, voluntary participation, consent requirements, and any other information quoted prior to each interview. The duration of each interview ranged from 30 to 45 min per participant. All interviews were recorded, and comprehensive notes were taken immediately afterward. The researchers began the data analysis concurrently with data collection after the first interview, transcribed the interviews with the participants’ permission, and continued until data saturation was achieved. Data collection took place over a period of 6 months from September 2023 to March 2024.

### 2.5 Trustworthiness and rigor

To ensure the trustworthiness and rigor of the study, the researchers considered credibility, transferability, dependability, and conformability following Braun and Clarke’s (35) framework. Before conducting the interviews, the researchers collaborated to establish criteria for participant selection and develop an interview guide with a focus on participant diversity. The researchers held frequent meetings to collectively review the transcripts until they reached a consensus on the voices of the participants. The researchers cross-checked each other’s work throughout the process to ensure accuracy in representing the findings. In addition, the researchers provided feedback to the interviewer after reviewing the transcripts to ensure an accurate reflection of the participants’ experiences. The participants did not make any alterations or suggestions in the transcripts. To improve transferability, the final report provides an elaborate account of the study methodologies and data, making the findings relevant to comparable populations or studies. A peer researcher conducted consistency checks to confirm agreement among the researchers on the accuracy, relevance, and meaning of the data, thereby ensuring reliability and conformability. Direct quotes from the interviewees supported the findings of the study.

### 2.6 Data analysis

To analyze the qualitative data, thematic analysis was employed following the framework established by Braun and Clarke (35). This approach is particularly well-suited for exploring participants’ lived experiences, offering a detailed understanding of the factors influencing program effectiveness, students’ job market preparedness, and areas for improvement. The systematic identification of recurring themes and patterns in the data provides valuable insights into the impact of career education programs and suggests ways to enhance their effectiveness.

Data collection and analysis were conducted iteratively starting after the initial interviews and continuing throughout the study. The analysis began with familiarization, in which the interviews were transcribed verbatim and reviewed multiple times. The initial codes were generated by identifying significant phrases and recurring themes. These codes were then organized into categories by examining the similarities and differences between the data. Finally, the themes were reviewed and refined to ensure that they accurately represented the data and addressed the research questions (35).

### 2.7 Ethical considerations

This study was approved by the CONJ Research Unit and KAIMRC under approval number NRJ23J/055/02, ensuring compliance with all ethical considerations regarding human participation. Prior to data collection, written consent was obtained from all participants, who were informed of their right to withdraw from the study at any time and that participation was voluntary. To maintain anonymity, participants were identified by numbers rather than names in the interview excerpts. The researchers also implemented strict confidentiality measures for the management and storage of study data, securely storing them on personal computers.

## 3 Results

### 3.1 Descriptive data of the interviewed participants

A total of 28 participants were successfully interviewed; half were senior nursing students at their fourth academic year and the other half were intern students enrolled in the internship year. The participants were predominantly 22 years old (17 participants), with smaller groups of 23 (five participants) and 25 (six participants). All interviewees attended the CGCP sessions. Twenty-three participants perceived the program as very effective, while five viewed it as fairly effective. None of the participants perceived it as neutral, very ineffective, or ineffective (see Table 2).

**Table 2:** Characteristics of the interviewed participants (*N* = 28).

Demographic data	No.	%
Age (years)		
22	17	60.7
23	5	17.9
25	6	21.4
Academic level		
Senior nursing students (fourth year)	14	50.0
Intern students	14	50.0
Perceived effectiveness of Career Guidance and Counseling Program		
Very effective	23	82.1
Fairly effective	5	17.9
Neutral/ Very ineffective/ Ineffective	0	0.00
Number of sessions attended in the GCCP program		
14 (all)	28	100.0

### 3.2 Thematic analysis framework

Table 3 provides an overview of the themes and categories derived from thematic analysis, encompassing seven main themes and 32 subthemes/categories. The main themes were personal experiences with the CGCP, the program’s importance, effectiveness, benefits, strengths, weaknesses, and recommendations for improvement. Sample quotations from participants are provided below, along with an explanation of each theme. Participants’ statements are denoted with codes using the letter “P” followed by a corresponding number indicating the interview order (e.g., P1, P2, P3).

**Table 3:** Emerged themes and categories from qualitative data analysis.

Themes (7)	Subthemes/factors (32)	Sample quotations for each factor
Theme I. Personal experience of the CGCP	Impressive and valuable personal experience Personal reflection on own experience	*“My experience with the program was very valuable. I gained a lot of knowledge that prepare me for my future career. The program is very effective in helping me understand the next steps in my life and how I can make my internship year a good experience.”* *“I found the experience to be wonderful and effective. The attendance of the career program motivated me to strive for success, reflect on my experience, and share it with peers. It was a great experience and a privilege to attend such a meeting for our beloved seniors and pass down the information to future generations.”*
Theme II. Importance of the CGCP	Career exploration and management Self-efficacy and confidence Career choice and certainty Self-awareness Career readiness Job market orientation Awareness of soft and hard skills Career and scholarship opportunities Sound career choice and realistic career goals. Smooth transition from education to employment.	*“The career guidance program really opened my eyes and explore various career paths I had not considered before, and it helped me manage my career planning more effectively.”* *“Participating in these sessions has greatly boosted my confidence and self-awareness, while also reducing my anxiety and uncertainty about the future”* *“The career guidance program has been instrumental in helping me to choose the right career path and make a well-informed career choice.”* *“The sessions have allowed me to gain a better understanding of my strengths and weaknesses, enabling me to make better decisions about my future.”* *“The program taught me how to create a strong resume and prepare for interviews, which makes me feel more ready to start my career.”* *“I was unsure about which area of nursing to specialize in, but through the sessions, I am oriented about different specialties and market and how to align them with my personal interests and strengths.”* *“Through the program, I have been able to develop necessary soft and hard skills and competencies required in the professional world.* *Also, the guidance program has offered me insights into further education, scholarship and training opportunities, both for self-development and professional growth.”* *“Of course I found this program important, and it provided me with valuable information about job prospects in different fields and has assisted me in setting realistic career goals.”* *“I now feel more prepared for the job market and have the necessary tools to smoothly transition from education to employment.”*
Theme III. Effectiveness of the CGCP	Very effective with positive impact on career readiness and job market	*“I found the program as 100% effective because it provides opportunities for networking and connecting with professionals in* var*ious fields, which can be very beneficial for career readiness and development among students.”* *“Overall, I believe that the program has a strong impact on helping students identify their career choices, set realistic goals and enhance their chances.”*
Theme IV. Benefits of the CGCP	Career transition Career path Knowledge of the labor market requirements Comprehensive knowledge of career exploration and career planning	*“The benefits of the received sessions are numerous, opening my eyes to a world of opportunities and advantages I never knew existed. They prepared me for the transition into my desired role and has given me confidence in my career choices.”* *“I never realized how important career guidance and counseling could be until I participated in the program. Now I know the different options for my career academic or clinical.”* *“The program taught me how to keep up with the constantly changing job market and gave me insight into what employers are looking for. This knowledge has been invaluable in helping me strategize my career path.”* *“Participating in the program allowed me to understand the needs of the labor market and work on developing myself to meet those needs. The program team was knowledgeable, and I gained valuable experience from them.”* *“I was pleasantly surprised to learn about the best hospitals and their advantages and disadvantages through the career guidance program. It’s important to know the industry you are entering, and this program provided me with that knowledge and allow me to start planning my career.”*
Theme V. Strengths of the CGCP	Well-organized program Variety of topics covered Quality of content and speakers’ approach Discovery of new knowledge Interaction among participants and speakers Interactive games (game-based-learning) The online provision of the program	*“I honestly could not find any weaknesses in the program. It was well-organized, and the speakers were helpful. I was satisfied with their assistance.”* *“The variety of topics covered in the program was fantastic. It gave me a well-rounded skill set in career guidance and counseling.”* *“I was really impressed by many sessions, such as the one on preparing for the Saudi licensure exam. It provided me with useful information and resources, which I believe will improve my chances of success.”* *“The content of the program and the quality of the sessions were excellent. It covered important and relevant topics in our field of work and help me to discover new knowledge.”* *“The speakers were really knowledgeable and provided great insights and guidance. I learned a lot from them and appreciate their approach in presenting the information and interacting with audience.”* *“This program exposed me to new knowledge that I wasn’t aware of before. It broadened my understanding of my career.”* *“I enjoyed the opportunity to interact with both the presenters and attendees. It fostered a lively and interesting environment.”* *“I believe that the inclusion of interactive games like Kahoot, MentiMeter, and Wordwall adds strengths to this program. Learning through fun and activities not only enhances participation, but also deepens our understanding and retention of key concepts.”* *“I value the option of providing this program through online sessions. My internship’s schedule program challenged me to attend physically, so being able to engage virtually assures that I can still benefit from the valuable content and discussions offered.”*
Theme VI. Weaknesses of the CGCP	The timing of the sessions The tight academic and clinical schedule Convenience to all students	*“I often had to choose between attending lectures and sessions. It was disappointing because I wanted to benefit from both, but the timing just did not work out with me.”* *“Managing my internship and attending counseling sessions was quite challenging. The schedule was so tight that sometimes I had to miss out on valuable guidance.”* *“I felt like the timing of the sessions was more aimed towards the senior students. I hoped for more flexibility so that everyone could participate.”*
Theme VII. Recommendations for Enhancing the CGCP	Flexible timing of the sessions Periodical provision of the program Adding more valuable speaker and sessions Learning need assessment of students. Feedback and improvement	*“I think that you should consider the timing of the sessions to ensure it is convenient for students and maximizes attendance.”* *“I think it would be helpful to schedule the career guidance and counseling sessions after lecture time so that more students can attend.”* *“It is important to continuously promote the program and encourage students to attend, so they can benefit from the career guidance and counseling.”* *“We acknowledge all the information presented in the sessions as valuable and beneficial to students. Adding more speakers and training sessions in the next cycle will be another strength.”* *“To cater to the diverse needs of students, you should offer more sessions covering different topics and areas of interest.”* *“Taking feedback from students and asking about the specific topics they need guidance on will help tailor the sessions to their needs.”*

#### 3.2.1 Theme I: personal experience of the CGCP

Participants described their experience with the CGCP as highly valuable, noting that it provided them with knowledge beneficial for their future careers. Many reflected that attending the program motivated them to strive for success and enabled them to pass on what they had learned to future generations. They stated that the program emphasized personal development and self-reflection and included activities and discussions that helped them better understand their interests, values, and passions. Overall, participants viewed the program as a transformative experience that prepared them for their careers and expressed gratitude for the opportunity to learn from professionals in the field.

*“My experience with the program was very valuable. I gained a lot of knowledge that prepare me for my future career. The program is very effective in helping me understand the next steps in my life and how I can make my internship year a good experience.” P2.*

*“I found the experience to be wonderful and effective. The attendance of the career program motivated me to strive for success, reflect on my experience, and share it with peers. It was a great experience and a privilege to attend such a meeting for our beloved seniors and pass down the information to future generations.” P5.*

#### 3.2.2 Theme II: importance of the CGCP

Overall, the students found the CGCP to be important. Students stated that it helped them become more self-aware of their strengths and interests as well as gain a better understanding of the essential soft and hard skills needed for success. The program also provided them with valuable information about various career and scholarship opportunities, allowing them to explore different paths and make informed decisions. Additionally, they stated that the program prepared students for the job market by offering insights into industry trends and demands and equipping them with essential career exploration and management skills. They also expressed a sense of certainty about their future paths, which helped alleviate their anxiety and uncertainty. Ultimately, the program facilitated a smooth transition from education to employment for students.

*“Of course I found this program important, and it provided me with valuable information about job prospects in different fields and has assisted me in setting realistic career goals.” P1.*

*“Participating in these sessions has greatly boosted my confidence and self-awareness, while also reducing my anxiety and uncertainty about the future.” P7.*

#### 3.2.3 Theme III: effectiveness of the CGCP

Participants reflected on the effectiveness of the CGCP, noting its positive impact on career readiness and the job market. They found the program to be highly effective and a significant influence on individuals’ lives and overall career development. The program provided valuable support, guidance, and resources, enabling the participants to make informed career decisions, overcome obstacles, and realize their full potential. As a result, the students felt more confident and capable of setting realistic goals and making sound career choices.

*“I found the program as 100% effective because it provides opportunities for networking and connecting with professionals in* var*ious fields, which can be very beneficial for career readiness and development among students.” P11.*

*“Overall, I believe that the program has a strong impact on helping students identify their career choices, set realistic goals and enhance their chances.” P2.*

#### 3.2.4 Theme IV: benefits of the CGCP

Participants reported that the CGCP was beneficial, highlighting career transition support, guidance in defining career paths, understanding labor market requirements, and gaining comprehensive knowledge of career exploration and planning.

*“The benefits of the received sessions are numerous, opening my eyes to a world of opportunities and advantages I never knew existed. They prepared me for the transition into my desired role and has given me confidence in my career choices.” P25.*

*“Participating in the program allowed me to understand the needs of the labor market and work on developing myself to meet those needs. The program team was knowledgeable, and I gained valuable experience from them.” P26.*

#### 3.2.5 Theme V: strengths of the CGCP

According to participants, the strengths of the CGCP include its well-organized structure and the variety of topics covered. Participants enjoyed all sessions, preparing for Saudi licensure exams and scholarship opportunities, and finding their way after the internship year. They found that the content quality and speakers’ approach were commendable and facilitated the discovery of new knowledge. They also valued the interaction between participants and speakers as well as the incorporation of interactive games for engaging in learning experiences. Additionally, participants praised the program’s online provision for accessibility and flexibility.

*“I believe that the inclusion of interactive games like Kahoot, MentiMeter, and Wordwall adds strengths to this program. Learning through fun and activities not only enhances participation, but also deepens our understanding and retention of key concepts.” P16.*

*“I value the option of providing this program through online sessions. My internship’s schedule program challenged me to attend physically, so being able to engage virtually assures that I can still benefit from the valuable content and discussions offered.”P12.*

*“I enjoyed the opportunity to interact with both the presenters and attendees. It fostered a lively and interesting environment.” P19.*

*“I was really impressed by many sessions, such as the one on preparing for the Saudi licensure exam. It provided me with useful information and resources, which I believe will improve my chances of success.” P24.*

#### 3.2.6 Theme VI: weaknesses of the CGCP

Some students identified several challenges, rather than weaknesses, in the CGCP, noting conflicting timing with their academic lectures, which caused inconveniences for certain students. Interns also mentioned challenges in managing schedules during internship shifts. Additionally, some students felt that the topics were more geared toward target senior students. However, it is worth noting that, despite these weaknesses, students expressed overall satisfaction with the program and did not perceive any significant drawbacks.

*“I often had to choose between attending lectures and sessions. It was disappointing because I wanted to benefit from both, but the timing just did not work out with me.” P4.*

*“I felt like the timing of the sessions was more aimed towards the senior students. I hoped for more flexibility so that everyone could participate.” P14.*

#### 3.2.7 Theme VII: recommendations for enhancing the CGCP

Participants offered several recommendations for improving CGCP such as implementing flexible session timings to accommodate their varying schedules and commitments better. They also proposed periodically offering the program to ensure continuous support and accessibility for students. Additionally, participants recommended enriching the program by incorporating more valuable speakers and sessions on diverse topics, particularly emphasizing the importance of conducting learning needs assessments to tailor the program to the students’ specific requirements. Finally, participants highlighted the need to establish feedback mechanisms to gather input for the ongoing improvement and enhancement of the program.

*“I think that you should consider the timing of the sessions to ensure it is convenient for students and maximizes attendance.” P25.*

*“Taking feedback from students and asking about the specific topics they need guidance on will help tailor the sessions to their needs.” P21.*

**Figure 2:**
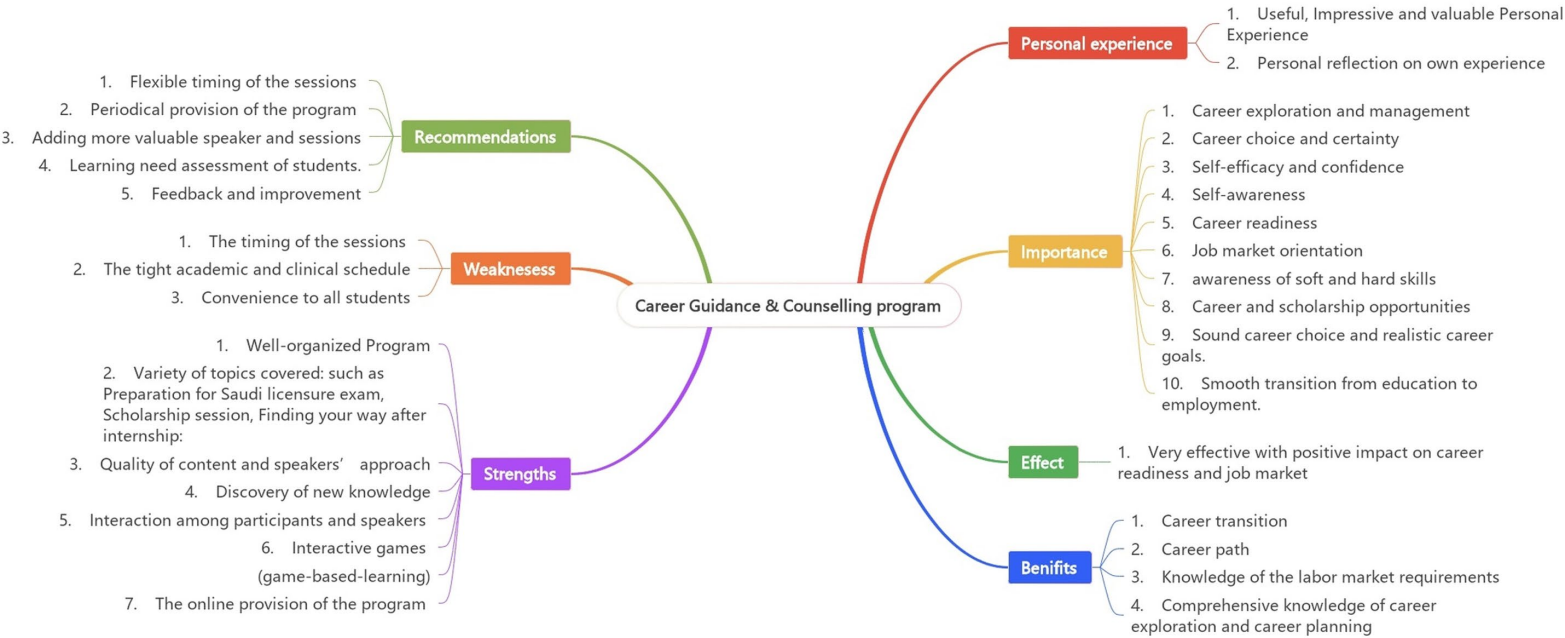
Summary of emerged themes and subtopics/categories.

See Figure 2 for the summary of emerged themes and categories.

## 4 Discussion

The CGCP plays a crucial role in shaping individuals’ professional paths by equipping them with the knowledge, skills, and confidence needed to navigate the complexities of the job market. This study explored the experiences and perspectives of student participants in a specific CGCP offered at a Saudi nursing college. By examining these perspectives, the researchers gained insights into the program’s effectiveness and its impact on students’ professional development and preparedness. Generally, our findings are supported by SCCT proposed by Lent et al. (14), which explains how career aspirations are influenced by cognitive processes (e.g., self-efficacy, outcome expectations) and external factors (e.g., social support). In our study, SCCT serves as a valuable framework for understanding how career guidance interventions impact students’ workforce readiness (Figure 1). The theory’s focus on self-efficacy, goal setting, and social support closely aligns with our findings, highlighting the critical role that these factors play in shaping career choices and interests (15, 16). This reinforces SCCT’s relevance in understanding students’ career preparations.

### 4.1 Participants’ personal experience and perceived importance of the CGCP

To answer the first and second research questions, the participants reflected on their personal experiences with the CGCP and underscored its important role in shaping their future careers. The nursing students found that the program provided them with valuable experiences, improved knowledge, and insights that would aid them in their professional journeys. The principle of experiential learning supports this positive feedback as participants actively engage with the material at a personal level, resulting in a deeper understanding of their interests, values, and career aspirations (12, 36). Additionally, they reflected that the program emphasized personal development and self-reflection, contributing to participants’ understanding of their interests, values, and passions. This emphasis on self-awareness aligns with existing research that highlights the importance of career guidance programs in facilitating career decision-making and preparing individuals for the workforce.

Furthermore, participants emphasized the importance of the CGCP in enhancing self-awareness, understanding essential skills, and preparing for the job market. This recognition of the importance of the program aligns with SCCT, which emphasizes the role of observation and direct experiences in enhancing career decision-making and self-efficacy (14). Moreover, participants expressed increased confidence and certainty about their career choices, indicating the importance of the program in empowering students to successfully navigate their career trajectories. These findings are consistent with studies emphasizing the significance of career guidance programs in providing information, resources, and psychological preparation to support career decision-making and adaptation to the work environment (37–39).

### 4.2 Effectiveness and benefits of the CGCP

In addressing the third and fourth research questions, participants reflected on the positive effects of the CGCP on their career readiness and development. The program provided valuable support, guidance, and resources that helped participants overcome obstacles and make informed career decisions. This result aligns with previous research indicating that well-designed career guidance programs significantly influence career decision-making, job search behaviors, and career satisfaction (37, 38, 40, 41).

Participants also highlighted benefits such as self-assessment, understanding the job market, gaining industry knowledge, and preparing for professional success, which aligns with previous findings (42, 43). Additionally, they reported benefits, such as career transition support and guidance in defining career paths, underscoring the holistic approach of career guidance programs in addressing various aspects of career development (44, 45). These findings are supported by studies on the effectiveness of career development courses (7, 46, 47). Abou Hashish (11) highlighted that career awareness among nursing students increases their self-efficacy by making them aware of their strengths and weaknesses. Thus, integrating the CGCP with experiential learning and psychological support empowers individuals to make informed career choices and achieve success.

### 4.3 Strengths and weaknesses of the CGCP

In response to the fifth question, participants identified several strengths of the CGCP, highlighting its well-organized structure, variety of topics covered, quality of content, speakers’ approaches, interactive learning activities, and online format. These strengths align with those of previous studies that emphasized best practices in career guidance programs, structured content delivery, engagement through interactive activities, and access to diverse resources and expertise (37). Moreover, participants appreciated the program’s online provision, noting its accessibility and flexibility, which are essential for accommodating students’ diverse schedules and preferences. The findings regarding the impact of online counseling on career decision-making are consistent with those of Rutten et al. (48) and Pordelan et al. (49), who investigated the effects of online training on career adaptability and development. Given the constraints on face-to-face career guidance services, online services offer advantages by reaching a larger number of students and accommodating their schedules (50). As the Internet becomes an integral part of daily life for people of all ages, serving as a platform for communication, entertainment, and information retrieval, the increasing reliance on technology presents opportunities for career counseling professionals to innovate and enhance their services (51).

While participants expressed overall satisfaction with the CGCP, they also identified several challenges they encountered during the program, including conflicting timing with academic lectures, difficulties in managing schedules with internship shifts, and the perceived focus of the sessions on senior students and the topics covered. These challenges underscore the importance of addressing participants’ recommendations to ensure program accessibility and quality.

### 4.4 Recommendations for improving the CGCP

In response to the final question, participants offered valuable recommendations for enhancing the CGCP, including implementing flexible session timings, offering the program periodically, enriching it with more valuable speakers and sessions, conducting student learning needs assessments, expanding the range of topics covered, and establishing feedback mechanisms for ongoing improvement. These recommendations align with previous findings that emphasize the importance of responsiveness to student needs, continuous improvement, and stakeholder engagement. For instance, Khurumova and Pinto (50) advocated accurate and regular assessments of college students’ needs to uphold the quality of the services provided. Additionally, Azhenov et al. (7) suggested enhancing university career counseling services through personalized one-on-one sessions to help students assess their strengths, weaknesses, and career goals. They emphasized the need for lifelong skill development in job seeking and career transitions, particularly as students approach their final years of undergraduate studies. In this context, Abou Hashish and Bajbeir (9) and Abou Hashish (11), recommended that nursing curricula continuously provide educational opportunities to assist nursing students in enhancing their career self-efficacy and talent development. This preparation is crucial for them to fulfill their professional roles effectively and overcome potential career barriers.

## 5 Study strengths and limitations

Although the CGCP received positive feedback from the participants, several limitations were identified. First, scheduling conflicts with academic lectures and internship shifts posed challenges for attendance and participation, potentially preventing some students from fully benefiting from the program. Additionally, some participants expressed concerns about the limited variety of topics covered, suggesting the need for broader content to address diverse interests and needs. Another limitation is its scope, which was confined to a single university, which raises questions about whether the institution’s specific characteristics influenced students’ traits and levels of engagement in career development sessions. Despite these limitations, this study highlights several strengths of career guidance programs. Participants appreciated the well-structured content delivery and engagement in activities such as games and discussions. They also praised the program for its online accessibility and flexibility, which allowed them to participate more conveniently.

## 6 Conclusion

This study aimed to evaluate the effectiveness and impact of the CGCP on students’ readiness for the job market, with a focus on several key questions, including nursing students’ personal experiences with the program; its perceived importance, effectiveness, and benefits; its strengths and weaknesses; and recommendations for improvement. Participants reported significantly positive experiences with the CGCP, highlighting its role in supporting career development and job market readiness, noting the program’s effectiveness in enhancing self-awareness, understanding essential skills, and preparing for future careers. The CGCP was particularly effective in bridging the gap between academic learning and practical job market requirements, resulting in increased confidence and a better understanding of career skills.

The strengths of the CGCP include its well-organized structure, quality content, speaker engagement, interactive learning activities, and online provision. However, participants also identified challenges, such as scheduling conflicts and limited topic coverage, which impacted their overall experience. These findings address the research questions regarding the program’s strengths and areas of improvement. Participants offered several recommendations for enhancing CGCP, including implementing flexible session timings, periodically offering the program, enriching it with more valuable speakers and sessions, conducting learning needs assessments, expanding topic coverage, and establishing feedback mechanisms. These suggestions align with the best practices in career guidance and emphasize the need for continuous improvement and responsiveness to students’ needs.

In summary, CGCP demonstrated considerable value in aiding career development and market readiness. The program’s strengths, particularly its content delivery and interactive approach, were evident in participants’ positive experiences. Addressing the identified limitations is crucial to further improve the program and ensure its continued success in preparing students for professional careers. By catering to the diverse needs of all participants and continuously refining the program based on feedback and assessments, the CGCP can better support students in their career development journey.

Based on the feedback and identified limitations, several implications can be made to enhance the quality and effectiveness of the CGCP.

### 6.1 Implications for nursing education

Nursing curricula should continue to provide more systematic and continuous career educational opportunities for nursing students and graduates to assist students in selecting career paths aligned with their interests and roles. Nurse educators should also acquire expertise to integrate innovative strategies into the curriculum to enhance students’ and graduates’ capacity to navigate and succeed at all stages of their career development.

For CGCP coordinators, it is recommended that flexible scheduling of sessions be implemented to accommodate participants’ diverse schedules, thereby reducing conflict with academic lectures and internship shifts. Additionally, diversifying the topics covered in the program will ensure inclusivity and relevance for all participants, thus addressing concerns regarding limited variety. Targeted outreach efforts should be developed to encourage junior students to participate and benefit from the program. Continuous improvement mechanisms (e.g., collecting participant feedback and conducting regular evaluations) should be established to identify areas of enhancement. Finally, stakeholder engagement with students, faculty, and industry professionals can help align the program with participants’ needs and expectations, ultimately strengthening its impact and effectiveness in empowering students to successfully navigate their career paths.

### 6.2 Implications for future research

To address the limitations of this study, further research is warranted to validate the effectiveness of the CGCP. This can be achieved through mixed method studies that integrate quantitative data on program effects from a larger sample size and qualitative feedback from participants’ perspectives. Replicating this study in a broader context and employing a true experimental design with both experimental and control groups could further validate the program’s effectiveness. In addition, longitudinal and curriculum-based career planning and development programs should be implemented. These programs should involve trained faculty career coaches and measure outcomes (e.g., career self-efficacy, career readiness, perceived career barriers) and other relevant factors.

## Data Availability

The raw data supporting the conclusions of this article will be made available by the authors, without undue reservation.
